# Characterization of tungiasis infection and morbidity using thermography in Kenya revealed higher disease burden during COVID-19 school closures

**DOI:** 10.1186/s40249-023-01080-5

**Published:** 2023-03-21

**Authors:** Lynne Elson, Abneel K. Matharu, Naomi Riithi, Paul Ouma, Francis Mutebi, Hermann Feldmeier, Jürgen Krücken, Ulrike Fillinger

**Affiliations:** 1 KEMRI-Wellcome Trust Research Programme, Hospital Road, Kilifi, Kenya; 2 Centre for Tropical Medicine and Global Health, Nuffield Department of Medicine, University of Oxford, Oxford, UK; 3 International Centre of Insect Physiology and Ecology, Human Health Theme, Nairobi, Kenya; 4 College of Veterinary Medicine, Animal Resources and Biosecurity, Makerere University, Kampala, Uganda; 5 Institute of Microbiology, Infectious Diseases and Immunology, Charité University Medicine, Berlin, Germany; 6 Institute for Parasitology and Tropical Veterinary Medicine, Freie Universität Berlin, Berlin, Germany

**Keywords:** Tungiasis, Neglected tropical diseases, Thermography, Morbidity, Child, COVID-19, Kenya

## Abstract

**Background:**

Tungiasis is a neglected tropical skin disease caused by the sand flea *Tunga penetrans*. Female fleas penetrate the skin, particularly at the feet, and cause severe inflammation. This study aimed to characterize disease burden in two highly affected regions in Kenya, to test the use of thermography to detect tungiasis-associated inflammation and to create a new two-level classification of disease severity suitable for mapping, targeting, and monitoring interventions.

**Methods:**

From February 2020 to April 2021, 3532 pupils age 8–14 years were quasi-randomly selected in 35 public primary schools and examined for tungiasis and associated symptoms. Of the infected pupils, 266 were quasi-randomly selected and their households visited, where an additional 1138 family members were examined. Inflammation was assessed using infra-red thermography. A Clinical score was created combining the number of locations on the feet with acute and chronic symptoms and infra-red hotspots.

**Results:**

The overall prevalence of tungiasis among all the school pupils who were randomly selected during survey rounds 1 and 3 was 9.3% [95% confidence interval (*CI*): 8.4–10.3]. Based on mixed effects logistic models, the odds of infection with tungiasis among school pupils was three times higher in Kwale (coastal Kenya) than in Siaya [western Kenya; adjusted odds ratio (*aOR*) = 0.36, 95% *CI*: 0.18–0.74]; three times higher in males than in females (*aOR* = 3.0, 95% *CI*: 2.32–3.91) and three times lower among pupils sleeping in a house with a concrete floor (*aOR* = 0.32, 95% *CI*: 0.24–0.44). The odds of finding an infected person among the household population during surveys before the COVID-19 pandemic was a third (*aOR* = 0.32, 95% *CI*: 0.19–0.53) of that when schools were closed due to COVID-19 restrictions and approximately half (*aOR* = 0.44, 95% *CI*: 0.29–0.68) in surveys done after school re-opening (round 3). Infection intensity was positively correlated with inflammation as measured by thermography (Spearman’s rho = 0.68, *P* < 0.001) and with the clinical score (rho = 0.86, *P* < 0.001). Based on the two-level classification, severe cases were associated with a threefold higher level of pain (*OR* = 2.99, 95% *CI*: 2.02–4.43) and itching (*OR* = 3.31, 95% *CI*: 2.24–4.89) than mild cases.

**Conclusions:**

Thermography was a valuable addition for assessing morbidity and the proposed two-level classification of disease severity clearly separated patients with mild and severe impacts. The burden of tungiasis was considerably higher in households surveyed during COVID-19 restrictions suggesting underlying risks are found in the home environment more than in school.

**Supplementary Information:**

The online version contains supplementary material available at 10.1186/s40249-023-01080-5.

## Background

Tungiasis is a highly neglected tropical skin disease (NTSD) caused by the female sand flea, *Tunga penetrans,* which penetrate the skin, usually of the feet, of their mammalian hosts and stay embedded for their remaining life [1]. The flea grows 2000-fold in size over seven days as a result of eggs developing in the abdomen. A small opening is maintained in the skin through which the last segments of the abdomen stay in contact with the environment. Via this opening, the male copulates with the embedded female [2] and the female expels eggs, respires, and defecates. Eggs fall to the ground and, if conditions are favorable, the larvae hatch and grow over several larval stages, pupate and emerge as adults over the course of 3–4 weeks [3]. The embedded female dies after egg-laying and is removed by skin repair mechanisms if not extracted by the host.

*Tunga penetrans* is endemic in the tropics of the Americas and in sub-Saharan Africa with an estimated 668 million people considered to be at risk of infection in sub-Saharan Africa alone [4, 5]. In endemic areas, tungiasis is heterogeneously distributed with the poorest part of the population bearing the highest burden [6]. In Kenya, human tungiasis is considered a significant individual and public health threat, with an estimated two million people currently infected [7] although there is no systematically collected data from national level surveillance. In general, males, children, elderly people and people with disabilities carry the highest disease burden [6] with prevalence ranging between 7 and 60% in affected villages and schools [8–10]. Intensity of infection is also heterogeneously distributed among the infected population, with the majority of patients having only a few embedded fleas, while a few individuals have over 100 fleas [9].

Sand fleas cause severe morbidity in humans, companion animals and livestock [11–13]. Morbidity results from the intense inflammatory response around the rapidly growing female sand fleas firmly embedded in the epidermis [11]. The inflammation is further intensified by frequent bacterial superinfection of the lesions and bacterial superinfection may result in tetanus, gangrene or septicemia [14].

A study conducted in Brazil 20 years ago carefully documented the clinical features of tungiasis [15]. Acute symptoms included itching, pain, edema, erythema, warmness, desquamation, ulcers and fissures. Chronic symptoms, thought to be the result of repeated infection with large numbers of fleas, included hyperkeratosis, peri-ungual hypertrophy, deformation and loss of nails [15]. The authors developed systematic methods to quantify the severity of disease with severity scores for acute tungiasis (SSAT) and chronic tungiasis (SSCT). Modified versions of these scores have also been applied to estimate morbidity in pigs and dogs and to quantify the effects of successful treatment on disease severity [16, 17].

Overall, the SSAT and SSCT have only rarely been used since they were published. One of the reasons may be that edema, erythema and warmness-to-touch, the signs of inflammation, are difficult to assess, particularly on a dark skin and by non-clinical staff. The SSAT also included scores for the number of sites with flea clusters, and the patient’s experience of pain, itching and sleep disturbance. It was demonstrated that those individuals with the highest infection rate (newly embedded live fleas over time) had the highest SSAT [15]. A separate study showed that the number of live fleas and the SSAT scores were negatively correlated with quality of life as measured using the modified Dermatological Quality of Life Index (mDQLI) [18].

Now that tungiasis has been added to the World Health Organization (WHO) list of NTDs (under scabies and other ectoparasites), governments and organizations will start to plan surveillance and intervention programs, and these will need carefully defined indicators and targets. As has been implemented for other NTDs, initially it will be appropriate to target those individuals with the highest morbidity. For instance, the WHO NTD Roadmap 2021–2030 [19] recommends targets using the prevalence of medium and high infection intensity for schistosomiasis and soil-transmitted helminths. The thresholds to define low, medium, and high intensity infection, such as eggs per gram of stool, were set by the WHO Technical Working Groups and were based on the morbidity caused by differing levels of infection intensity [19–21]. Similar measures and thresholds will be needed for tungiasis.

To date, disease intensity for tungiasis has been classified in three-tiers, defined using embedded flea counts; mild cases being defined as having 1–5 fleas, moderate cases having 6–30 fleas and severe cases more than 30 fleas [9, 10, 22]. However, the first studies describing this classification did not explain the reasoning behind the thresholds and they have not been associated with levels of morbidity. As has happened for some other NTDs whose targets focus on medium and high intensity disease [23], i.e. reducing the classification from three groups to two, it may be more appropriate to define two disease groups for tungiasis rather than three.

Consequently, in the current study we set out to (i) describe the prevalence and intensity of infection in two regions of Kenya with suspected high disease burden; (ii) simplify the clinical scoring method; (iii) test the use of thermography to assess inflammation; (iv) evaluate the past disease severity classification with respect to symptoms in these populations in Kenya and develop a new classification with only two disease severity groups. The data reported here is part of a larger project aimed at characterizing the disease ecology of tungiasis in East Africa, including a better understanding of the parasite, risk factors for severe tungiasis and its impact on child development and well-being.

## Methods

### Study design

A cross-sectional observation study of children in primary schools, their families, and homesteads in two regions of Kenya was carried out between February 2020 and April 2021. Primary school children between the age of 8 and 14 years were selected since this is the age group most affected by tungiasis in Kenya [22].

### Study area and population

**Fig. 1:**
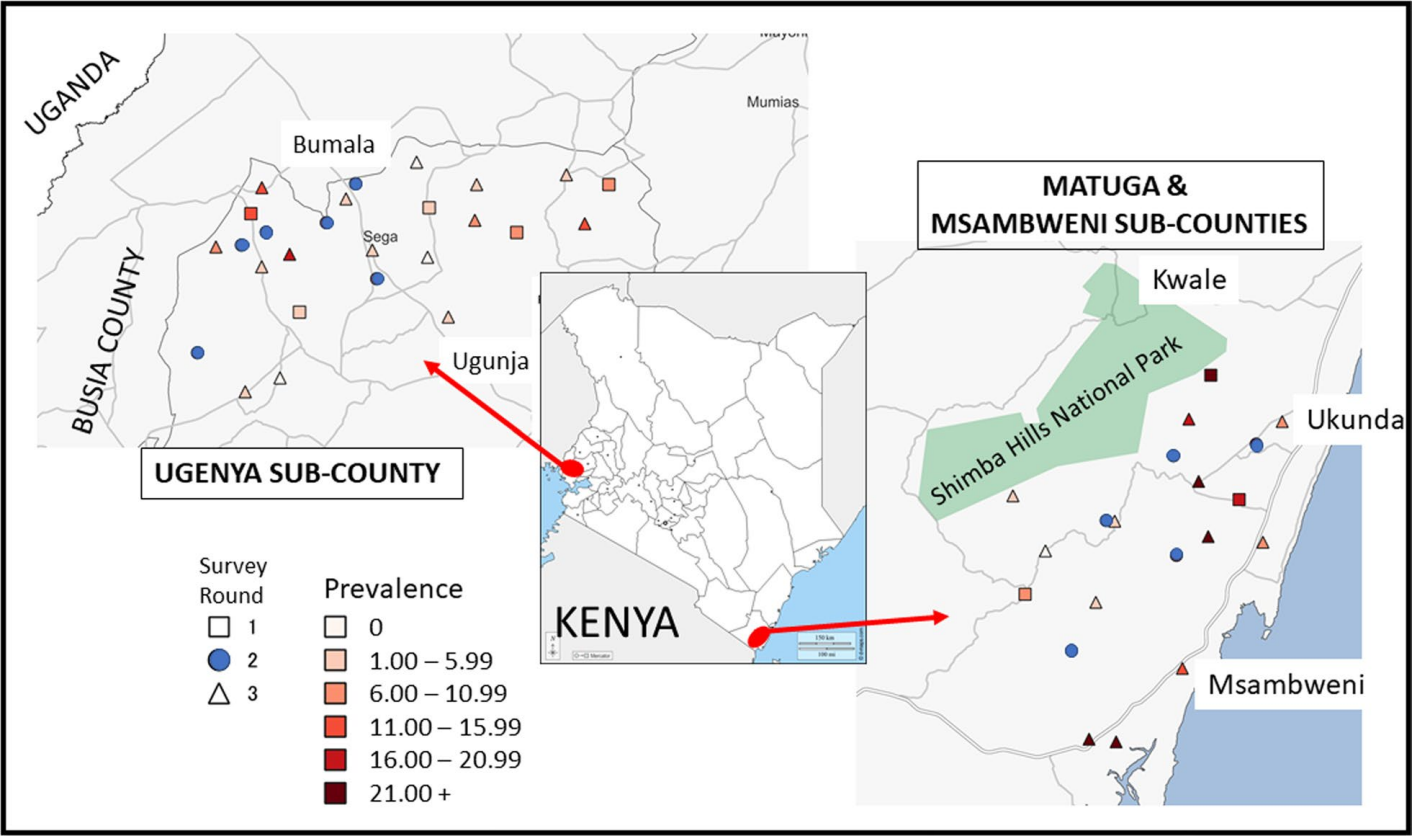
Map of the study sites to show the location in Kenya, schools and catchment areas by survey round and the prevalence of tungiasis in the schools during round 1 and 3 (squares and triangles, respectively). Prevalence is not indicated for round 2 catchment areas surveyed during COVID-19 restrictions (blue circles)

Surveys, following the same procedures, were conducted in the sub-counties of Matuga and Msambweni of Kwale county on the south east coast of Kenya, and in Ugenya sub-county of Siaya county in western Kenya near the border with Uganda (Fig. 1). These counties were chosen since they lie in similar ecological zones but are inhabited by people with different ethnicity, cultures and livestock-keeping habits. Both counties are major areas of sugar cane-production and thus a proportion of the population is engaged in this agro-industry. Both areas were among the counties with the highest tungiasis prevalence rate according to the Kenyan Ministry of Health in 2014 [7].

Average rainfall amount (mm) for each month of the study from October 2019 to April 2021 was retrieved from World Weather Online [24] for the nearest possible sites with available data; Siaya town near Ugenya sub-county and Matuga town in Kwale. For data analyses the average rainfall for the two months prior to the survey month was calculated, for example the average rainfall in mm for April and May for all households visited in June. Both measures, average rainfall in the month of the visit and average rainfall in the two months before the visit, were converted from mm to cm (by dividing by 10) of rainfall for ease of interpretation of the outcomes from regression analyses.

### Sampling procedure

Within Kwale and Siaya counties, sub-counties were selected that were known by the county Department of Health to have a high tungiasis burden. Lists of all existing public primary schools in the sub-counties were provided by the county education departments and 35 schools randomly selected using a paper lottery approach. In each school, 51 boys and 51 girls between the age of 8 and 14 years (the age with the highest prevalence and intensity of tungiasis [22]) were quasi-randomly selected by lining the pupils up into three age groups (8 and 9 years; 10 and 11 years; 12–14 years) and by sex within each age group. Every n^th^ (depending on the total number in the group) pupil was then selected in each sex and age group until 17 from each was reached, to a total of 102. All selected pupils were examined for tungiasis, and a small questionnaire was administered asking about the floor of the house they sleep in and whether other people in the family are infected. The examinations and interview were conducted by field enumerators trained and supervised by the authors. Out of all tungiasis infected pupils identified in a school, a maximum of ten who reported living in a home with an unsealed soil or sand floor were randomly (paper lottery method) selected for household surveys. If only ten or less than ten were identified in a school, all were selected. We specifically targeted households with unsealed floors since this risk factor has been well-established for tungiasis [8, 25, 26] and for our risk factor study to be reported elsewhere we wanted to explore other determinants of disease. This strategy was used from February to mid-March 2020.

COVID-19 restrictions forced the closure of schools from March to December 2020. Field research activities were however free to recommence from July 2020. Consequently, the survey strategy was adapted to recruit cases through a household-based survey only. In the catchment areas of 11 schools already selected randomly before COVID-19 closures, community health volunteers (CHVs) were asked to invite any children aged 8 to 14 years they considered to be infected, to a location where they could be screened by the study team. Out of all infected children seen through this approach, a maximum of ten were randomly selected for household surveys. This survey strategy was used between August and October 2020 (survey round 2) returning to school-based surveys in January to May 2021 (survey round 3). No school or household was visited more than once. Figure 1 shows the location of the schools screened in round 1 and 3 and the catchment areas in round 2, demonstrating their equal distribution across the study area.

### Clinical assessment procedures

The clinical assessment procedures were the same for the pupils in schools and for the household members of the selected pupils.

The feet of the 102 children in each school were washed and dried and systematically examined for the presence of tungiasis by observing nine zones on each foot in order from the largest toe to the smallest toe, the medial side, lateral side, the sole and the heel. Those pupils found to have sand fleas embedded in the feet, were assessed for intensity of infection by counting the number of fleas that were: alive (round white lesion with dark spot at center); dead (black, irregular-shaped lesion); manipulated (lesion from where a flea had clearly been removed) or large clusters of embedded fleas in which individual fleas could not be counted as they were too close together.

Infected individuals were also assessed for acute and chronic symptoms by modifying the technique described by Kehr et al. [15]. Both feet were examined systematically by nine zones each (five toes, medial side, lateral side, sole and heel). For each of the zones, the presence or absence of each symptom was recorded. These included desquamation, fissures, ulcers and abscess for acute symptoms and hyperkeratosis, peri-ungual hypertrophy, deformed nails and lost nails for chronic symptoms. In comparison to the original description of the score by Kehr et al. [15], the signs of inflammation; edema, erythema and warmness, were not recorded due to the difficulty of assessing these for non-clinicians.

Infected individuals were also asked to report the amount of pain and, separately, the amount of itching they felt in their feet associated with the embedded fleas using the options: “none at all”, “a little”, “some” or “a lot”.

### Infra-red thermography

**Fig. 2:**
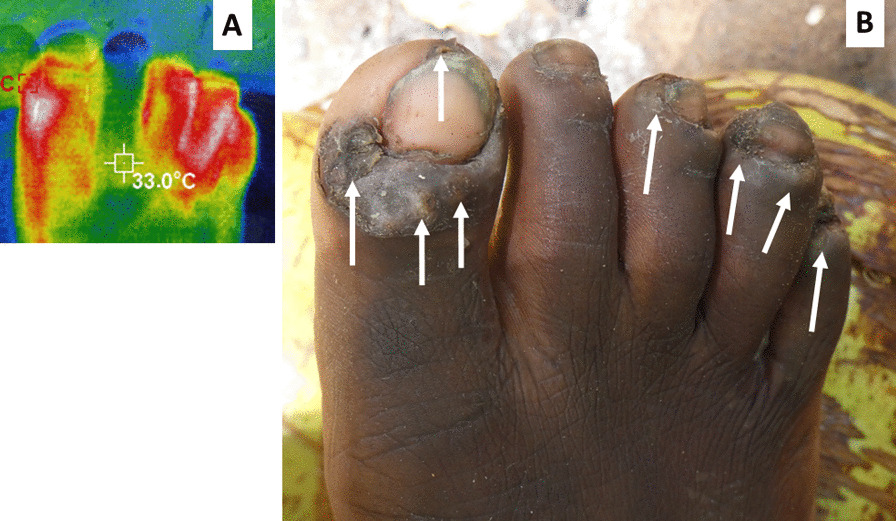
Infrared thermography of an infected foot showing four sites (toes) with inflammation associated with embedded fleas. Infra-red image with rainbow transformation (**A**). Normal photograph of the same foot (**B**). White arrows indicate sites with hotspots (white) associated with embedded live fleas

A low-resolution (220 × 160) infra-red camera detecting wavelengths of 8–14 µm (Hti Thermal Imaging Camera, HT-A1, Dongguan Xintai Instrument Co. Ltd, Dongguan, Guangdong Province, China) was used to observe the nine zones, both upper and lower surfaces of both feet. These cameras convert the different temperatures detected into a range of colors (Fig. 2) The hottest being white or yellow in the rainbow spectra transformation used. The presence or absence of such a “hotspot” was recorded for each of the 18 zones on the feet. If there was more than one hotspot in a zone it was recorded as one site. Elevated temperature as detected by thermography around embedded fleas, has been shown to be associated with acute tungiasis [27].

### Sample size

The number of pupils screened per school was based on the assumption that we would find a maximum of 10% of the pupils in any given school infected. With an average school size of 300 children in the target age group, disease prevalence of 10% surveys would require screening at least 95 pupils to achieve 80% power and 95% confidence. To allow for pupils to opt out, we randomly selected 102 in each school. The number of clusters surveyed over the study duration was largely driven by another research question on risk factors which will be reported elsewhere.

### Data analyses

All analyses were conducted in Stata IC version 15.1 (Stata Corp LLC, College Station, TX, USA). For the pupil and household populations surveyed in schools and homes, prevalence (proportion of examined participants) and 95% confidence intervals (*CI*) were calculated for geographic regions, sexes, age groups and survey round. To test for associations between tungiasis infection status (infected and not infected) and geographic region, sex, age, survey round and rainfall (independent variables), multilevel mixed effects (MLME) logistic models were used with an exchangeable correlation matrix. The unique school ID was included as a random effect for the pupil population and the household unique ID for the household population. Initially, bivariable analyses were run for each independent variable and then multivariable analysis with all variables. Backward elimination was used to develop the final model using Akaike information criteria (AIC) to compare goodness of fit of the models. Bivariable outcomes are presented as odds ratios (*OR*) and multivariable outcomes as adjusted odds ratios (a*OR*) calculated as the exponential of the coefficient with 95% *CI* and* P*-values.

For analysis of infection intensity and associated symptoms, a new dataset was created merging all infected participants from both school and household-based surveys. The infection intensity for each infected individual was calculated as the sum of all flea types (live, dead and manipulated) on both feet, plus the number of flea clusters multiplied by 5 (as an estimate for an average number of fleas/cluster). Since infection intensities were not normally distributed, medians and interquartile ranges (IQR) were calculated for schools, regions, sexes, age groups and survey round. Since infection intensity was a positively skewed count variable for cases only, with no zeros, associations with region, sex, age, survey round and rainfall were tested using MLME linear regression models with zero-truncated negative binomial probability distributions and log link functions. The unique school ID was included as a random effect. Backward elimination was used to develop the final model using AIC to compare the goodness of fit of multivariable models. Bivariable outcomes are presented as incidence rate ratios (IRR) and multivariable outcomes and adjusted incidence rate ratios (AIRR) with 95% *CI* and *P*-values.

The correlation of school level prevalence and median infection intensity was tested using linear regression setting the intercept at zero since median infection intensity can only be zero if prevalence is zero. The coefficient is presented as R^2^ and the* P*-value.

Acute symptoms scores were calculated by summing the number of zones on the feet exhibiting each symptom, and then the scores for desquamation, fissures, ulcers and abscess were totaled, with a maximum possible score of 72 (9 zones × 2 feet × 4 pathologies). Chronic symptoms scores were calculated by summing the number of zones on the feet exhibiting each symptom and then the scores for hyperkeratosis, peri-ungual hypertrophy, deformed nails and lost nails totaled, with a maximum of 38 (10 for peri-ungual hypertrophy, 10 for either deformed nails or lost nails, 18 for hyperkeratosis). This was different to the previously described method of Kehr et al. [15] who assigned a score of 0.5, 1, 2 or 3 based on the number of sites affected for each symptom. We then went on to add the acute and chronic scores together in a ‘Total Symptoms’ score with a maximum of 110.

For the thermography, the infra-red hotspots were considered as a symptom and the total number of zones on the feet with a hotspot was totaled for each patient, with a maximum possible value of 18. Correlations were explored between infra-red scores, the three symptoms scores (acute, chronic, total) and intensity of infection using Spearman rank correlation coefficient since these variables were positively skewed and are presented as rho values in a correlation matrix. Once the infra-red scores were seen to correlate with the other variables, they were added to create a new clinical score.

To evaluate the association of the old disease intensity groups based on flea counts with the new clinical score, bivariable MLME linear models were used with negative binomial probability distributions, log link functions and exchangeable correlations. The unique school ID was included as a random effect. Outcomes are presented as incidence rate ratios (IRR) with 95% *CI* and *P*-values. To identify a possible new two-level classification for disease severity, a scatter plot was created of infection intensity by clinical score for all infected cases. The clinical scores were used as these were a measure of overall morbidity and reflect actual disease severity. The nonparametric Loess regression technique was used to fit a smooth curve through the scatter plot to help see the relationship between the two variables. The previous threshold levels for defining three levels of disease severity based on infection intensity alone [9, 10, 22] are marked at infection intensity of 5 and 30. Pain and itching levels were coded as 0 (none to a little) or as 1 (“some” to “a lot”). To assess the association of the new disease severity groups with the pain and itching levels MLME logistic models with an exchangeable correlation matrix and unique school ID was random effect were used.

## Results

### Pupil participants

**Fig. 3:**
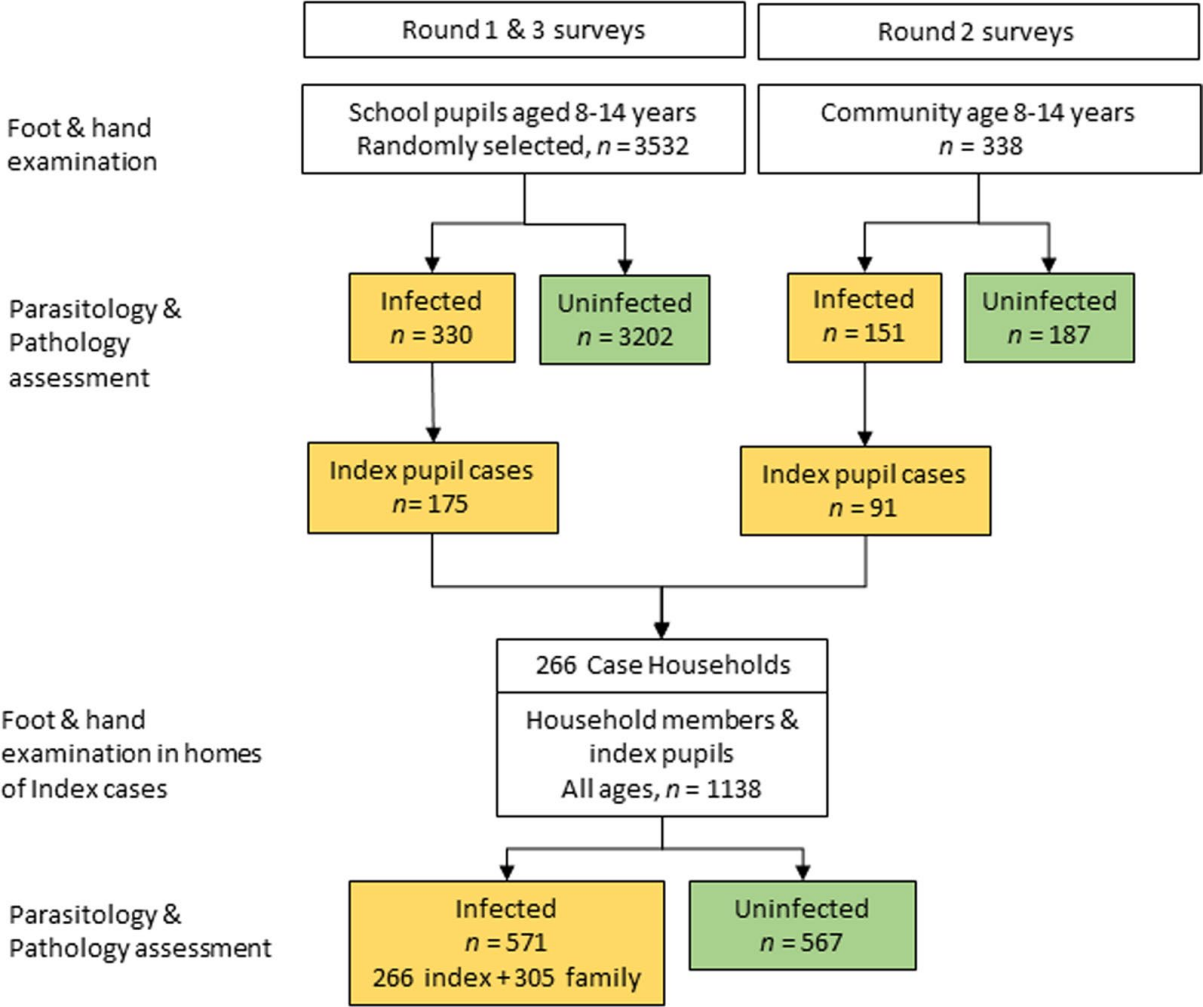
Flow diagram describing selection of participant groups. Orange boxes represent people infected with tungiasis, green boxes represent people who were not infected

Children age 8–14 years were screened for tungiasis in 35 primary schools, 15 schools in Msambweni/Matuga (Kwale) and 20 in Ugenya (Siaya), of which 8 were completed in 2020 (survey round 1) and the remainder in 2021 (survey round 3) when schools re-opened after the COVID-19 shut-down (Fig. 3; Table 1; Additional file 4). In total we examined 3535 pupils in schools, 810 in 2020 and 2723 in 2021. All raw data are provided in Supplementary Materials. As targeted, there were equal numbers of boys and girls enrolled, and the median age was 11 years (IQR: 9–12). In total, 62.5% of pupils reported they sleep in a house with a mud or sand floor and the remaining 37.5% have a concrete floor. In survey round 2, during COVID-19 school closures, 338 children aged 8 to 14 years were clinically examined in the communities, 207 in Kwale and 131 in Siaya. During community-based surveys in round 2, infected children age 8–14 years were specifically asked to attend a screening event and all attendees were examined. There was no randomized selection of the children so a true prevalence for those communities could not be assessed. Consequently, in line with expectations, more males than females were examined, given that risk of infection is usually higher in boys than girls.

**Table 1:** Study population examined for tungiasis. Number of individuals in each group

Survey round	1	2	3	Total
Months	Feb–Mar 2020	Aug–Oct 2020	Jan–Apr 2021	
**Pupils (8–14 years)**				
No. schools	8	0	27	35
No. pupils	810	338	2723	3871
Region				
Kwale	3/302	207	12/1224	15/1733
(# schools/# pupils)				
Siaya	5/508	131	15/1499	202,138
Sex (#pupils)				
Female	402	143	1363	1908
Male	408	195	1360	1963
Age (years, median, IQR^a^)	11 (9–12)	11 (9–12)	11 (9–12)	11 (9–12)
House floor				
Sand/mud	604	328	1606	2538
Concrete	206	10	1117	1333
**Households of selected pupils**				
# households	55	91	120	266
# occupants reported	378	611	867	1856
# individuals examined (%)	242 (64.0)	401 (65.6)	495 (57.1)	1138 (61.3)
Region				
Kwale	128	245	346	719
Siaya	114	156	149	419
Sex				
Female	122	202	261	585
Male	120	199	234	553
Age groups (years)				
< 8	49	94	114	257
8–14	96	170	178	444
15–21	19	31	27	77
22–41	44	66	92	202
42–60	20	26	59	105
> 60	14	14	25	53
Age (median years, IQR)	12 (8–30)	11 (8–24)	12 (8–33)	12 (8–30)

#, Number

^a^IQR: Interquartile range

### Household participants

A total of 266 households of infected pupils, living in a house with an unsealed sand or mud floor, were recruited for the study (Fig. 3; Table 1; Additional file 5). Within these households, respondents reported having a total of 1856 occupants (a mean of 7 people per household) of whom 1138 people were examined for tungiasis (61.3%, a mean of 4 people per household), the 266 pupils and 872 of their family members. The proportion available for examination at each survey round ranged from 57.1% in round 3 to 65.6% in round 2.

### Rainfall

**Fig. 4:**
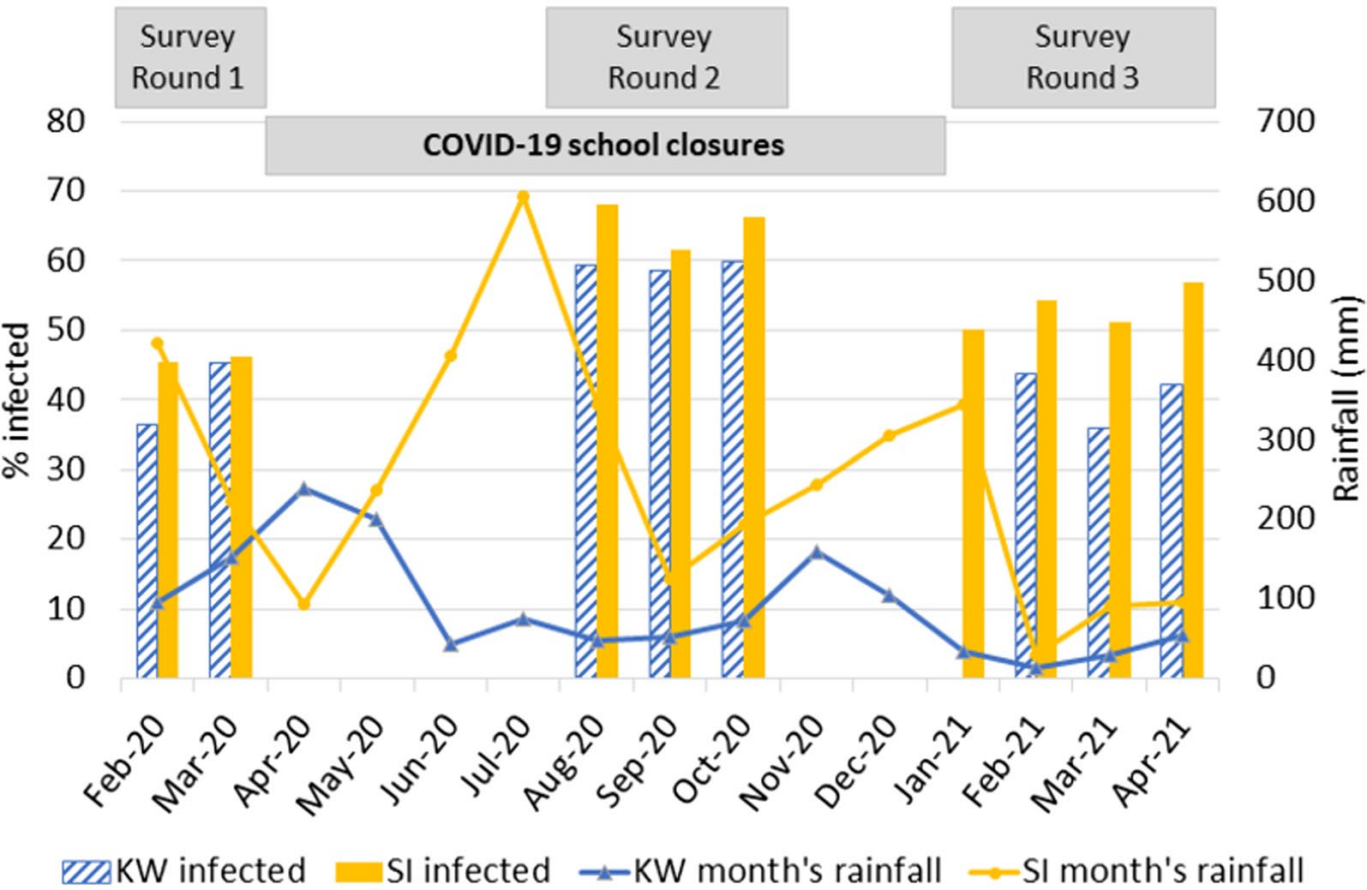
Proportion of household population infected by month and the monthly rainfall in Kwale (KW) and Siaya (SI)

Figure 4 illustrates the study area in western Kenya (Ugenya, Siaya) had higher rainfall than the location in coastal Kenya (Kwale) for most of the study period, and the surveys were mostly conducted during the drier months in both regions.

### Prevalence of tungiasis

The overall prevalence of tungiasis among all the school pupils who were randomly selected during survey rounds 1 and 3 was 9.3% (95% *CI*: 8.4–10.3). In multivariable models, boys had a three-fold higher odds of infection than girls (*aOR* = 2.9 and 3.0, Table 2) and there was a negative association between infection and age (*aOR* = 0.89), even within the narrow age range of examined pupils, 8–14 years. Pupils in Siaya had a nearly three-fold lower odds of infection (*aOR* = 0.36) than those in Kwale, as did pupils who sleep in a house with an unsealed sand or mud floor compared to those with a concrete floor (*aOR* = 0.36). There was no association with rainfall. The maps in Fig. 1 illustrate the prevalence of infection in the surveyed schools for round 1 and 3 and school catchment areas for round 2. From this map it appears that in both regions the schools with highest prevalence are locally clustered.

**Table 2:** Prevalence of tungiasis

Infection status	*N*^a^	Actual % infected	95% *CI*		Bivariable				Multivariable			
					*OR*^b^	95% *CI*		*P*	*a*OR^c^	95% *CI*^*d*^		*P*
**School pupils**												
Total	3532	9.3	8.4	10.3								
County												
Kwale	1526	13.6	11.9	15.4	1				1			
Siaya	2007	6.1	5.2	7.3	0.42	0.21	0.86	0.018	0.36	0.18	0.74	0.005
Sex												
Female	1765	5.4	4.4	6.5	1				1			
Male	1768	13.3	11.8	15	2.85	2.20	3.68	< 0.001	3.01	2.32	3.91	< 0.001
Age (years)					0.91	0.85	0.97	0.003	0.89	0.83	0.95	0.001
House floor												
Sand/mud	2209	12.1	10.8	13.5	1							
Concrete	1323	4.8	3.7	6.1	0.36	0.26	0.47	< 0.001	0.32	0.24	0.44	< 0.000
Survey round												
1	810	11.2	9.2	13.6	1							
3	2723	8.8	7.8	9.9	0.57	0.23	1.42	0.226				
Rainfall (cm) same month^e^					1.01	0.97	1.04	0.719				
Rainfall (cm) previous 2 months					1.01	0.96	1.06	0.603				
**Household Members of Index Cases**												
Total	1138	50.3	47.4	53.2								
County												
Kwale	719	47.1	43.5	50.8	1							
Siaya	419	55.6	50.8	60.3	1.4	1.1	1.79	0.006				
Sex												
Female	585	35.9	32.2	39.9	1				1			
Male	553	65.3	61.2	69.1	3.51	2.71	4.54	< 0.001	2.38	1.71	3.33	< 0.001
Age group (years)												
< 8	257	54.5	48.3	60.5	0.19	0.13	0.29	< 0.001	0.21	0.14	0.31	< 0.001
8–14	444	82.7	78.9	85.9	1				1			
15–20	77	23.9	15.4	35.2	0.04	0.02	0.07	< 0.001	0.04	0.02	0.08	< 0.001
21–40	202	9.9	6.4	14.8	0.01	0.01	0.02	< 0.001	0.02	0.01	0.03	< 0.001
41–60	105	10.1	5.7	17.3	0.01	0.01	0.03	< 0.001	0.02	0.01	0.04	< 0.001
60	53	30.2	19.4	43.8	0.06	0.03	0.12	< 0.001	0.07	0.03	0.15	< 0.001
Survey round												
1	242	42.3	36.2	48.7	0.46	0.33	0.64	< 0.001	0.32	0.19	0.53	< 0.001
2	401	61.3	56.5	66.0	1				1			
3	495	45.2	40.8	49.6	0.59	0.4	0.68	< 0.001	0.44	0.29	0.68	< 0.001
Rainfall (cm) same month^e^					1.01	1.0	1.02	0.004				
Rainfall (cm) previous 2 months					1.01	0.99	1.03	0.128				

^a^*N*: number of individuals

^b^*OR*: odds ratio

^c^*aOR*: adjusted odds ratio

^d^*CI:* confidence interval

^e^Rainfall unit in cm for ease of interpretation, converted from mm by dividing rainfall in mm by 10

By selection criteria, all the 266 households selected had at least one child age 8–14 years infected and an unsealed sand or mud floor. The overall prevalence in these households was 50.2% (95% *CI*: 47.4–53.2). As among the pupils, males had a higher odds of infection than females (*aOR* = 2.4, Table 2) when adjusted for other covariables. Children under 8 years had five-fold lower odds of infection than 8–14-year-old children (*aOR* = 0.21), but adults and children over 14 years had 50 to 100-fold lower odds of infection compared to the youngest age group.

When adjusted for sex and age, households examined during round 1 had a three-fold lower odds (AOR 0.33) of being infected than those seen in round 2, and households seen in round 3 had a more than two-fold lower odds of infection to those in round 2 (*aOR* = 0.44). The map in Fig. 1, shows that areas surveyed during round 2 were distributed evenly among those in round 1 and 3. There was no association of rainfall with prevalence of infection in the household population, so this difference between the survey rounds was unlikely to have been caused by changes in rainfall.

### Intensity of infection

To investigate infection intensity and the symptoms caused by embedded fleas, data for all infected individuals were combined (Additional file 6). Since there were very few infected individuals in the age groups older than 14 years (*n* = 44), they were excluded from analyses. Of the remaining 633 individuals with full data sets, 406 (64.1%) were from Kwale, 430 (67.9%) were male and 503 (79.5%) were age 8–14 years.

The infection intensity for the 633 cases ranged from 1 to 154 fleas, with a median intensity of 13 fleas (IQR: 5–28) (Table 3). Infection intensity among patients was associated with the region, sex and survey round with no change between bivariable and multivariable outcomes. Infected children in Siaya had a lower infection intensity than those in Kwale (*aIRR* = 0.67) which goes hand in hand with the overall lower prevalence in that region. Boys had a higher infection intensity than females (*aIRR* = 1.27) and those examined in survey round 1 (*aIRR* = 0.5) and 3 (*aIRR* = 0.63) had lower infection intensity than those in round 2. There was no association between infection intensity with age nor rainfall.

**Table 3:** Intensity of Infection of all cases (*n* = 633)

Intensity score	*N*^a^	Median	IQR^b^		Bivariable				Multivariable			
			25%	75%	*IRR*^c^	95% *CI*^*d*^		*P*	*aIRR*^e^	95% *CI*		*P*
Region												
Kwale	406	14	6	32	1				1			
Siaya	227	10	3	23	0.68	0.52	0.90	0.006	0.67	0.55	0.82	< 0.001
Sex												
Female	203	10	4	27	1				1			
Male	430	14	6	29	1.21	1.03	1.41	0.019	1.27	1.08	1.49	0.004
Age group (years)												
< 8	130	11	5	22	1							
8–14	503	14	5	31	1.22	0.96	1.54	0.104				
Survey round												
2	272	18	9	38	1				1			
1	42	7	2	20	0.49	0.27	0.92	0.026	0.50	0.32	0.79	0.003
3	319	10	4	20	0.64	0.49	0.83	0.001	0.63	0.51	0.77	< 0.001
Rainfall cm same month^f^	633	13	5	28	0.99	0.98	1.00	0.139				
Rainfall cm previous 2 months	633	13	5	28	0.98	0.97	1.00	0.115				

^a^*N*: number of individuals

^b^IQR: interquartile range

^c^*IRR*: incidence rate ratio

^d^*CI:* confidence interval

^e^*aIRR*: adjusted incidence rate ratio

^f^Average rainfall for the two months prior to the month a household was visited. Rainfall unit in cm for ease of interpretation, converted from mm by dividing rainfall in mm by 10

### Correlation of school prevalence and median infection intensity

**Fig. 5:**
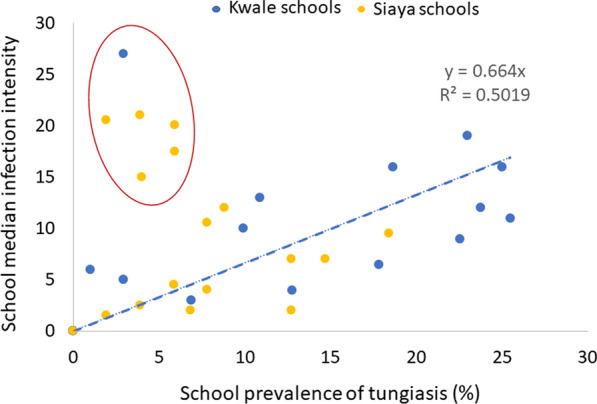
Scatter plot of school prevalence and median infection intensity score. Each dot represents one school. Dashed blue line represents the linear regression line with the intercept set at 0, its coefficient and R^2^ stated for all schools

The school prevalence ranged from 0% in four schools to 26% in one school (Fig. 5), with more than one third of schools having a prevalence higher than 10%. Using simple linear regression, there was a significant positive correlation between prevalence and median infection intensity in the schools (R^2^ = 0.502, *P* < 0.001). However, the scatter plot in Fig. 5 highlights six schools (in the circle) that did not fit well in this relationship. These schools were characterized by overall low prevalence of tungiasis in the pupil population but with the few infected pupils having a very high infection intensity.

### Tungiasis associated symptoms

Of the 633 infected participants, 598 (94.5%) were recorded to have at least one symptom. The most common was desquamation (88%), considered to be an acute symptom, followed by deformed nails (74%) and peri-ungual hyperkeratosis (58%) which are classified as chronic symptoms (Additional file 1). The least common were lost nails (21%), and abscesses caused by secondary bacterial infection (27%). The number of sites affected by each individual symptom was positively correlated with the number of live and dead fleas and the infection intensity (all *P* < 0.001), the strongest correlation being for infection intensity score with desquamation and deformed nails (rho 0.84 and 0.68 respectively, Additional files 2 and 3). The median score for the acute symptoms (desquamation, fissures, ulcers, abscess) using our simplified scoring system was 6 (IQR: 2–12) while that for chronic symptoms (hyperkeratosis, peri-ungual hyperkeratosis, deformed nails, lost nails) was 5 (IQR: 1–11). Using Spearman correlation, both the acute and chronic symptom scores were each strongly correlated with the infection intensity score (rho 0.818 and 0.766, respectively) and with each other (rho = 0.857, Table 4). A new score was introduced combining the acute and chronic symptoms scores into a single total symptoms score which had a median of 12 (IQR: 4–23) and had a slightly higher correlation with infection intensity than the two individual symptoms scores (rho 0.822, Table 4).

**Table 4:** Spearman rank correlation for symptom scores and intensity of infection (*n* = 633)

Spearman ($\uprho$)^a^	Infection intensity	Acute symptoms score	Chronic symptoms score	Total symptoms score	Infra-red score	Clinical score (with IR)
Infection intensity	1.0000					
Acute symptoms score	0.818	1.000				
Chronic symptoms score	0.766	0.857	1.000			
Total symptoms score	0.822	0.963	0.961	1.0000		
Infra-red score	0.684	0.562	0.477	0.541	1.000	
Clinical score (with IR^b^)	0.861	0.959	0.939	0.986	0.66	1.000

^a^All $\uprho$ values level of significance *P* < 0.001

IR: infra-red, had at least one hotspot indicative of inflammation

### Infra-red thermography

**Fig. 6:**
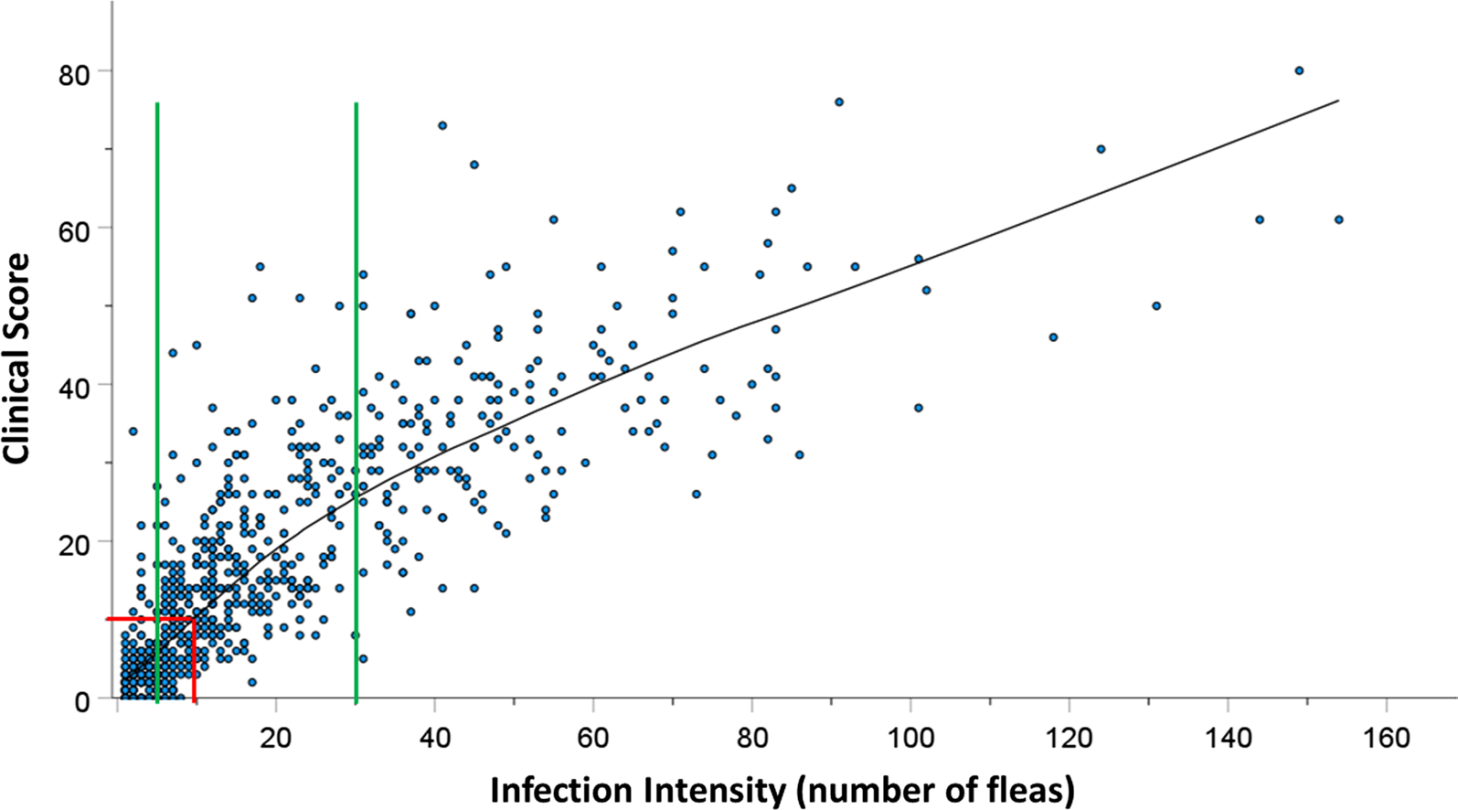
Scatterplot of infection intensity and clinical scores with Loess regression line fitted. Green lines indicate the past thresholds (5 and 30 fleas) used to define mild, moderate and severe disease. Red lines indicate the clinical score threshold of 10 and interpolation to 10 fleas.

Figure 2 illustrates this well, where a patient has many fleas and other symptoms but to the naked eye, inflammation is not obvious for a non-clinician. The accompanying thermograph, however, shows there was inflammation in four toes. In this study we have attempted to simplify the thermography by using more affordable cameras and simply counting the number of sites on the feet with hot spots. The median number of sites with a hotspot per patient was 2 (IQR: 0–5) and the highest number of hotspots was 17 of the maximum 18 sites. The number of infra-red hotspots was correlated with the infection intensity (rho = 0.684, Table 4), the acute symptoms scores (rho = 0.566), the chronic symptoms scores (rho = 0.477) and the total symptoms scores (rho = 0.541). Adding the infra-red scores to the total symptoms scores created a score with the strongest correlation to the infection intensity (rho = 0.861), the combination has been termed as ‘clinical score’. For this reason, the clinical score was considered the strongest variable to represent overall symptoms and its correlation to infection intensity is illustrated in Fig. 6.

### Classifying disease severity

Using the previously published classifications of disease severity based on infection intensity [9] as mild (1–5 fleas), moderate (6–30 fleas) and severe (> 30 fleas), of the 633 cases under 15 years, 172 (27.2%) were mild, 311 (49.1%) were moderate and 150 (23.7%) were severe. Bivariable models found individuals with moderate disease, had four-fold higher clinical scores than those with mild disease, and individuals with severe disease had nine-fold higher clinical scores (Table 5).

**Table 5:** Bivariable regression for clinical scores by various disease severity groups

Clinical scores	*N*^a^	Median (IQR^b^)	IRR^c^	95% *CI*^*d*^		*P*
Intensity-based groups						
Mild	172	3 (1–5)	1			
Moderate	311	15 (9–22)	4.1	3.6	4.7	< 0.001
Severe	150	36 (28–43)	9.3	8.1	10.7	< 0.001
Symptoms-based groups						
Mild	252	4 (2–7)	1			
Severe	381	25 (16–34)	6.2	5.6	6.8	< 0.001

^a^*N*: Number of individuals

^b^IQR: Inter-quartile range

^c^IRR: Incidence rate ratio

^d^*CI:* Confidence interval

To explore whether the previous threshold of five fleas was appropriate to create the two groups; mild with 1–5 fleas and severe with more than five fleas, a scatterplot of infection intensity by clinical scores (Fig. 6) was created. This revealed the five-flea threshold bisected a tight cluster of cases with low infection intensity and low clinical scores, placing some of them in the moderate disease category. Based on this scatterplot, we suggest a single threshold based on the clinical score of 10, at the upper limit of the low clinical score cluster would be a better fit for this group of children. A Loess trendline was fitted to the data to visually estimate a clinical score of 10 was equivalent to 10 fleas (red lines in Fig. 6). Using this proposed new classification, 381 (60.2%) of the 633 cases had severe disease and had six-fold higher clinical scores than individuals in the new mild category (*OR* = 6.2, Table 5).

Children with severe disease according to the proposed new classification, had a three-fold higher odds of reporting higher levels of pain and itching than those with mild disease (Table 6).

**Table 6:** Association of pain and itching with the revised disease severity classification

	*N*^a^	% with higher level	*OR*^b^	95% *CI*^*c*^		*P*
Pain						
Mild disease	231	20.9	1			
Severe disease	360	43.0	2.99	2.02	4.43	< 0.001
Itching						
Mild disease	231	25.2	1			
Severe disease	360	45.8	3.31	2.24	4.89	< 0.001

^a^*N*: Number of individuals

^b^*OR*: odds ratio

^c^*CI*: confidence interval

## Discussion

This study set out to characterize tungiasis in two different regions of Kenya, to simplify pathology assessments, to test the use of a thermographic method to detect inflammation, to evaluate the previously developed disease severity classification and to develop a new two-level classification of disease severity that could be used to target and monitor interventions in future, as has been done for other NTDs. The study was disrupted by the COVID-19 pandemic which caused the closure of schools throughout the country for approximately 10 months. Research activities resumed in August 2020, but schools were still closed, forcing a change in recruitment strategy for our study until schools re-opened in January 2021. Inadvertently, this enabled the study to identify what appears to be an unexpected indirect impact of COVID-19.

The proportion of the household population who were infected during surveys implemented when the schools had been closed for 5 months, was triple that seen in surveys done prior to school closure and double in surveys done when schools reopened. There could be several possible explanations for this observation. Screening children age 8–14 years in the village rather than pupils of the same age attending school, may have enrolled some who would not normally attend school and who may come from families with a higher risk of infection. However, the subsequent selection procedure of households to visit were the same in all three survey rounds, being random selection (paper lottery method) of ten infected children from all infected children age 8 to 14 years identified during screening.

Another explanation could be that the infected households visited during round 2 may always have had a higher household prevalence than those visited during round 1 and 3. Since the schools catchment areas targeted in round 2 are evenly distributed among those surveyed in round 1 and 3, and the schools and their catchment areas targeted in all rounds were all selected randomly, this is unlikely. Longitudinal cohort studies of schools and households may be one study design to avoid this, but the results of subsequent visits run the risk of being biased as the families become more aware of disease transmission and change behavior. In addition, research ethics compel researchers to provide access to treatment for cases which is likely to alter subsequent prevalence.

Changes in weather patterns could have been responsible since low rainfall has been shown to be associated with higher prevalence previously [28]. However, inclusion of rainfall in the multivariable models found no such association.

The higher prevalence during school closure could also have been caused by the extended time at home and the different behaviors during that time. During school closures children were spending all day at home or at homes of friends and relatives, resulting in more exposure to transmission sites per unit of time compared to spending most of the day in school classrooms. This would corroborate our previous finding [8] that children are at higher risk of infection at home than at school where they spend most of the day on concrete classroom floors. When not attending school, children were also less likely to wear shoes (personal observations). Shoe-wearing has been associated with lower risk in some studies [10, 26, 29] but not in others [30, 31]. This possibility will be explored in our later risk factor study.

The infection intensity among infected children was also significantly higher during school-closure. Since infection intensity was positively correlated with clinical scores and higher levels of pain and itching, this means that school closures due to COVID-19 resulted in more tungiasis-related suffering in children.

Our study has confirmed previous findings of the heterogeneity of tungiasis distribution [8, 22] with the prevalence in schools ranging from 0 to 18% in Siaya and from 1 to 26% in Kwale. In the current study, the overall prevalence among pupils in Siaya of 6.2% was significantly lower than in Kwale of 13.6% and lower than any previously published prevalence in schools [8, 32] or households [9, 10, 22, 29, 33] from targeted endemic areas. This difference in prevalence is likely due to many factors, including the fact that the current study randomly selected schools within sub-counties known to have tungiasis patients, while others specifically only targeted schools known to have pupils with tungiasis. Other possible causes could be the different age ranges included in the different surveys, differences in climate, cultures, and economic status of the community, and control efforts which may have been implemented prior to surveys. Indeed, in Siaya the survey teams were told that the county government and partners had been conducting intervention programs in some parts of the sub-county in the preceding year.

This study corroborates findings from past surveys from Brazil [34], Cameroon [35] and Kenya [22, 29], that males and children aged between 8 and 14 years have the highest prevalence, followed by those under 8 years and those over 60 years. The reasons for this sex and age distribution have yet to be determined but may be due to the different exposure and hygiene behaviors of boys, particularly in the 8–14-year age group [8]. Boys of this age tend to receive less attention from their caregivers, often must stop sleeping/living in their caregiver’s house and have to care for themselves [36]. It is also possible that in spending time together in close proximity in a crowded classroom or elsewhere, these children could infect each other since free-living, host-seeking female fleas have been shown to move between hosts [31]. The higher prevalence among the youngest and oldest members of the community may also reflect their inability to remove penetrating fleas with a sharp instrument [22]. The higher odds of infection among pupils with unsealed sand or mud floors was also as expected from past studies[22, 25, 26, 29] and likely reflects the developmental needs of the off-host stages and high odds of the emerging adults finding a suitable host.

As might be expected, the intensity of infection in school pupils was positively correlated to the prevalence, albeit with some outlier schools. The more children are infected in a community, the more contamination of the environment with off-host stages and the higher the exposure of others to infection. Even if most transmission is happening within homes [8], children of this age group often spend time visiting and even sleeping in the homes of friends and family, particularly during school holidays/closures (personal observations), and thus might be exposed to infection from other households. Further enquiries of the research team regarding the outlier schools where there was a prevalence of less than 7% but a high median intensity, found that in three of the six schools, two of the cases were siblings and some from families who were members of religious sects which do not accept any modern health care. In addition, these schools had received tungiasis interventions in the recent past and it is not inconceivable that these children/families refused treatment at that time, as they did when our study teams visited. Explaining these anomalies will probably require in-depth anthropological studies.

While some past studies have described tungiasis as a highly aggregated disease where the majority of cases had an intensity of infection of 1–5 fleas and only a few cases had a high intensity of infection with more than 30 fleas [9, 10, 22, 35], the current study found the majority of cases (47%) had 6–30 fleas, and 18% had more than 30 fleas. Our median intensity of infection of 13 among all infected individuals was also considerably higher than reported for other studies, which varied from a median of 2.5 in Cameroon [35] to 6 in Nigeria [33]. This suggests an overall higher burden of disease in the current study, and yet there was a lower prevalence of disease compared to past studies. This difference may have been caused by our intensity of infection measure incorporating a count of five for every flea cluster, and not a straight flea count. Other studies do not mention how flea clusters were incorporated in the flea counts, but possibly the enumerators attempted to count the closely packed fleas, which we felt was unlikely to be accurate in our circumstances.

Our simplified symptoms score is appropriate since both the acute and chronic symptoms scores correlated significantly with the infection intensity, and with each other. The most commonly reported symptom in previous studies was deformed nails, being as high as 98% of cases in a study focusing only on severe cases [15] and was seen on 73% of cases in the present study and correlated with infection intensity. Toenail loss and deformity are likely caused by fleas embedded in the nail bed and hyponychium causing direct physical damage as well as damage through inflammation. Some nail deformity may be the result of lost nails regrowing from damaged nailbeds. This suggests many of the cases in this study have been heavily infected for some time since deformed nails are the result of chronic and severe infection. This is striking since toenail loss and deformity are permanent if the nailbed is damaged and may remain as a mark of past disease and a source of stigma, shame, and discrimination for life. In fact, as part of a previous shoe donation program one of the investigators (LE) has had teenage girls explicitly say they are happy to receive shoes as they enable them to hide their toenails deformed by past infections.

To further simplify the assessment of inflammation we adapted the methodology of Schuster et al. [27] who demonstrated high resolution thermography can identify areas of inflammation by taking measurements of the temperature of the skin around embedded fleas and comparing that to other areas of the foot. Simple handheld infra-red cameras are affordable (costing as little as USD 170 for smart phone attachments or USD 385 for the model used in the current study) are readily available and can be used to locate areas of the skin that are hotter than others through a color transformation of the raw data. Instead of measuring temperatures of the skin we trained observers to record presence or absence of hot spots in the 18 zones of the feet used to record symptoms. The facts that the infra-red images revealed inflammation where no edema or erythema was visible as in Fig. 2 and that the number of sites with hot spots correlated significantly with the intensity of infection and the acute symptoms scores, suggest it is a good proxy measure for inflammation. Simple thermography such as this will likely be very useful in clinical trials to monitor the impact of treatment without the need of expensive equipment.

As governments and organizations begin to map tungiasis in their countries and to implement intervention programs, it will be important to have clear definitions for identifying target populations and intervention goals. Previously the three-tier classification of disease severity has been used, based only on flea counts with no description of how this correlated with symptoms. Since WHO guidelines for the control of other NTDs use targets based on two-tier levels of morbidity caused by different infection intensities, we propose a two-tier classification for tungiasis based on the Clinical score of 10. As with other NTDs, assessment of symptoms is time consuming, and parasite counts quicker and simpler to conduct for large scale public health projects, so we recommend a total flea count of 10 is an appropriate threshold for severe disease.

This study had three main limitations. While the pandemic accidentally afforded the opportunity to observe the impact of school closures, it also meant we could not continue the randomized sampling of pupils in all communities that was needed to obtain prevalence estimates across all sampling sites. Secondly, school-based sampling means the study would have missed children from the poorest families who cannot afford to send their children to school and given the association of tungiasis with poverty [6], possibly the most affected children. In addition, the survey would have missed those children who may have been unable to walk to school on account of having severe tungiasis. However, this was likely to have a minimal impact on the study outcomes since some children with high intensity infections were identified in the schools. Lastly, the study did not assess other measures of inflammation such as oedema, erythema and warmness of skin to compare with the thermographic measure.

## Conclusions

Tungiasis is a highly heterogeneous disease with the prevalence in schools varying considerably. Prevalence of tungiasis was positively correlated with infection intensity and with morbidity. Simplified thermography is a valuable addition for assessing morbidity associated with tungiasis and will be useful to assess the efficacy of treatment in future clinical trials. Along with other pathologies, thermography helped to classify mild and severe disease which will be used in our future studies on the impact of tungiasis. Fortuitously, the survey spanned the COVID-19 school closures and demonstrated that when children spent an extended period out of school, the prevalence, intensity and morbidity of tungiasis increased significantly indicating prevention measures and education should target household level infrastructure and behavior.

## Supplementary Information

**Additional file 1: **Frequency of acute and chronic symptoms associated with tungiasis. The number and percent of patients with acute and chronic symptoms.

**Additional file 2: **Spearman Correlation of individual flea counts and tungiasis associated acute symptoms. Description: a spearman correlation matrix with rho and p-value for each flea type count (live, dead, manipulated and clusters) and the number of sites on the feet with each of the acute symptoms (desquamation, ulcers, fissures, abscess).

**Additional file 3: **Spearman Correlation of individual flea counts and tungiasis associated chronic. Description: a spearman correlation matrix with rho and p-value for each flea type count (live, dead, manipulated and clusters) and the number of sites on the feet with each of the chronic symptoms (hyperkeratosis, peri-ungual hyperkeratosis, deformed nails, lost nails)

**Additional file 4: **Elson Pupil dataset.

**Additional file 5: **Elson Households dataset.

**Additional file 6: **Elson Child cases dataset.

## Data Availability

The datasets supporting the conclusions of this article are available in the supplementary materials associated with this manuscript.

## Abbreviations

SSAT: Severity scores for acute tungiasis
SSCT: Chronic tungiasis
mDQLI: Modified Dermatological Quality of Life Index
*OR*: Odds ratio
*CI*: Confidence interval
AIC: Akaike information criteria
IRR: Incidence rate ratios

## References

[1] Eisele M, Heukelbach J, Van Marck E, Mehlhorn H, Meckes O, Franck S (2003). Investigations on the biology, epidemiology, pathology and control of Tunga penetrans in Brazil: I. Natural history of tungiasis in man. Parasitol Res..

[2] Lynne Elson MT, Fillinger U, Feldmeier H. Replication data for: infection with tungiasis through inter-host movement of adult female sandfleas, tunga penetrans. Harvard Dataverse. 2020. 10.7910/DVN/E6IFU1.

[3] Nagy N, Abari E, D'Haese J, Calheiros C, Heukelbach J, Mencke N (2007). Investigations on the life cycle and morphology of Tunga penetrans in Brazil. Parasitol Res.

[4] Deka MA (2020). Mapping the geographic distribution of tungiasis in Sub-Saharan Africa. Trop Med Infect Dis.

[5] Heukelbach J, de Oliveira FA, Hesse G, Feldmeier H (2001). Tungiasis: a neglected health problem of poor communities. Trop Med Int Health.

[6] Feldmeier H, Heukelbach J, Ugbomoiko US, Sentongo E, Mbabazi P, von Samson-Himmelstjerna G (2014). Tungiasis—a neglected disease with many challenges for global public health. PLoS Negl Trop Dis.

[7] Ministry of Health. National policy guidelines on prevention and control of jigger infestations. Nairobi, Kenya: Division of Environmental Health; 2014. http://guidelines.health.go.ke/#/category/12/95/meta

[8] Elson L, Wiese S, Feldmeier H, Fillinger U (2019). Prevalence, intensity and risk factors of tungiasis in Kilifi County, Kenya II: results from a school-based observational study. PLoS Negl Trop Dis.

[9] Muehlen M, Heukelbach J, Wilcke T, Winter B, Mehlhorn H, Feldmeier H (2003). Investigations on the biology, epidemiology, pathology. II. Prevalence, parasite load and topographic distribution of lesions. Parasitol Res..

[10] Girma M, Astatkie A, Asnake S (2018). Prevalence and risk factors of tungiasis among children of Wensho district, southern Ethiopia. BMC Infect Dis.

[11] Feldmeier H, Eisele M, Saboia-Moura RC, Heukelbach J (2003). Severe tungiasis in underprivileged communities: case series from Brazil. Emerg Infect Dis.

[12] Mutebi F, Krücken J, Feldmeier H, Waiswa C, Mencke N, Sentongo E (2015). Animal reservoirs of zoonotic tungiasis in endemic rural villages of Uganda. PLoS Negl Trop Dis.

[13] Heukelbach J (2005). Tungiasis. Rev Inst Med Trop Sao Paulo.

[14] Feldmeier H, Heukelbach J, Eisele M, Sousa AQ, Barbosa LM, Carvalho CB (2002). Bacterial superinfection in human tungiasis. Trop Med Int Health.

[15] Kehr JD, Heukelbach J, Mehlhorn H, Feldmeier H (2007). Morbidity assessment in sand flea disease (tungiasis). Parasitol Res.

[16] Mutebi F, von Samson-Himmelstjerna G, Feldmeier H, Waiswa C, BukekaMuhindo J, Krucken J (2016). Successful treatment of severe tungiasis in pigs using a topical aerosol containing chlorfenvinphos, dichlorphos and gentian violet. PLoS Negl Trop Dis.

[17] Dos Santos KC, Chiummo RM, Heckeroth AR, Zschiesche E, Brandão Guedes PE, Harvey TV (2022). Efficacy of oral fluralaner (Bravecto) against Tunga penetrans in dogs: a negative control, randomized field study in an endemic community in Brazil. PLoS Negl Trop Dis.

[18] Wiese S, Elson L, Feldmeier H (2018). Tungiasis-related life quality impairment in children living in rural Kenya. PLoS Negl Trop Dis.

[19] World Health Organization (2020). Ending the neglect to attain the Sustainable Development Goals: a road map for neglected tropical diseases 2021–2030.

[20] Wiegand RE, Secor WE, Fleming FM, French MD, King CH, Deol AK (2021). Associations between infection intensity categories and morbidity prevalence in school-age children are much stronger for *Schistosoma haematobium* than for *S. **mansoni*. PLoS Negl Trop Dis..

[21] Malizia V, Giardina F, de Vlas SJ, Coffeng LE (2022). Appropriateness of the current parasitological control target for hookworm morbidity: a statistical analysis of individual-level data. PLoS Negl Trop Dis.

[22] Wiese S, Elson L, Reichert F, Mambo B, Feldmeier H (2017). Prevalence, intensity and risk factors of tungiasis in Kilifi County, Kenya: I. Results from a community-based study. PLoS Negl Trop Dis..

[23] Levecke B, Cools P, Albonico M, Ame S, Angebault C, Ayana M (2020). Identifying thresholds for classifying moderate-to-heavy soil-transmitted helminth intensity infections for FECPAKG2, McMaster, Mini-FLOTAC and qPCR. PLoS Negl Trop Dis.

[24] World Weather Online. World Weather Averages 2022. https://www.worldweatheronline.com.

[25] Muehlen M, Feldmeier H, Wilcke T, Winter B, Heukelbach J (2006). Identifying risk factors for tungiasis and heavy infestation in a resource-poor community in northeast Brazil. Trans R Soc Trop Med Hyg.

[26] Ugbomoiko US, Ariza L, Ofoezie IE, Heukelbach J (2007). Risk factors for tungiasis in Nigeria: identification of targets for effective intervention. PLoS Negl Trop Dis.

[27] Schuster A, Thielecke M, Raharimanga V, Ramarokoto CE, Rogier C, Krantz I (2017). High-resolution infrared thermography: a new tool to assess tungiasis-associated inflammation of the skin. Trop Med Health.

[28] Heukelbach J, Wilcke T, Harms G, Feldmeier H (2005). Seasonal variation of tungiasis in an endemic community. Am J Trop Med Hyg.

[29] Nyangacha RM, Odongo D, Oyieke F, Bii C, Muniu E, Chasia S (2019). Spatial distribution, prevalence and potential risk factors of Tungiasis in Vihiga County, Kenya. PLoS Negl Trop Dis.

[30] Thielecke M, Raharimanga V, Rogier C, Stauss-Grabo M, Richard V, Feldmeier H (2013). Prevention of tungiasis and tungiasis-associated morbidity using the plant-based repellent Zanzarin: a randomized, controlled field study in rural Madagascar. PLoS Negl Trop Dis.

[31] Elson L, Thielecke M, Fillinger U, Feldmeier H (2022). Infection with tungiasis through interhost movement of adult female sand fleas, Tunga penetrans. Trans R Soc Trop Med Hyg.

[32] Mwangi J, Ozwara H, Gicheru M (2015). Epidemiology of tunga penetrans infestation in selected areas in Kiharu constituency, Murang’a County, Kenya. Trop Dis Travel Med Vacc..

[33] Ugbomoiko US, Ifeanyi Ofoezie E, Heukelbach J (2007). Tungiasis: high prevalence, parasite load, and morbidity in a rural community in Lagos State, Nigeria. Int J Dermatol.

[34] Wilcke T, Heukelbach J, Cesar Saboia Moura R, Regina Sansigolo Kerr-Pontes L, Feldmeier H (2002). High prevalence of tungiasis in a poor neighbourhood in Fortaleza, Northeast Brazil. Acta Trop.

[35] Collins G, McLeod T, Konfor N, Lamnyam C, Ngarka L, Leo N (2009). Tungiasis: a neglected health problem in rural cameroun. Int J Collaborat Res Intern Med Public Health.

[36] Mojola SA (2014). Providing women, kept men: doing masculinity in the wake of the African HIV/AIDS epidemic. Signs (Chic).

