# Supplementary material for: Characterization of tungiasis infection and morbidity using thermography in Kenya revealed higher disease burden during COVID-19 school closures
Source: Infect Dis Poverty. 2023 Mar 21;12:24. doi: 10.1186/s40249-023-01080-5 (PMC10027580; doi:10.1186/s40249-023-01080-5)
Supplement: Supplementary file 1 — Additional file 1: Frequency of acute and chronic symptoms associated with tungiasis. The number and percent of patients with acute and chronic symptoms. [file 40249_2023_1080_MOESM1_ESM.docx]

## Additional File 1 Frequency of acute and chronic symptoms associated with tungiasis

**Characterization of tungiasis infection and morbidity using thermography in Kenya revealed increase in disease burden during COVID-19 school closures.**

Lynne Elson^1,2, *^, Abneel K. Matharu^3^, Naomi Riithi^3^, Paul Ouma^3^, Francis Mutebi^4^, Herman Feldmeier^5^, Jürgen Krücken^6^, Ulrike Fillinger^3^

^1^ KEMRI-Wellcome Trust Research Programme, Kilifi, Kenya

^2^ Centre for Tropical Medicine and Global Health, Nuffield Department of Medicine, University of Oxford, United Kingdom.

^3^ International Centre of Insect Physiology and Ecology, Human Health Theme, Nairobi, Kenya

^4^ College of Veterinary Medicine, Animal Resources and Biosecurity, Makerere University, Kampala, Uganda

^5^ Institute of Microbiology, Infectious Diseases and Immunology, Charité University Medicine, Berlin, Germany

^6^ Institute for Parasitology and Tropical Veterinary Medicine, Freie Universität Berlin, Germany

## Frequency of acute and chronic symptoms associated with tungiasis

|  | Number of patients with symptom | % of patients with symptom |
| --- | --- | --- |
| ACUTE |  |  |
| Desquamation | 559 | 88.8 |
| Infrared hotspots | 462 | 73.0 |
| Fissures | 271 | 42.8 |
| Ulcers | 216 | 34.1 |
| Abscess | 170 | 26.9 |
| CHRONIC |  |  |
| Deformed nails | 469 | 74.1 |
| Peri-ungual hyperkeratosis | 365 | 57.7 |
| Hyperkeratosis | 334 | 52.8 |
| Lost nails | 133 | 21.0 |
