# Supplementary material for: Characterization of tungiasis infection and morbidity using thermography in Kenya revealed higher disease burden during COVID-19 school closures
Source: Infect Dis Poverty. 2023 Mar 21;12:24. doi: 10.1186/s40249-023-01080-5 (PMC10027580; doi:10.1186/s40249-023-01080-5)
Supplement: Supplementary file 3 — Additional file 3: Spearman Correlation of individual flea counts and tungiasis associated chronic. Description: a spearman correlation matrix with rho and p-value for each flea type count (live, dead, manipulated and clusters) and the number of sites on the feet with each of the chronic symptoms (hyperkeratosis, peri-ungual hyperkeratosis, deformed nails, lost nails) [file 40249_2023_1080_MOESM3_ESM.docx]

## Additional File 3. Spearman ccorrelation of individual flea counts and tungiasis associated chronic symptoms

**Characterization of tungiasis infection and morbidity using thermography in Kenya revealed increase in disease burden during COVID-19 school closures.**

Lynne Elson^1,2, *^, Abneel K. Matharu^3^, Naomi Riithi^3^, Paul Ouma^3^, Francis Mutebi^4^, Herman Feldmeier^5^, Jürgen Krücken^6^, Ulrike Fillinger^3^

^1^ KEMRI-Wellcome Trust Research Programme, Kilifi, Kenya

^2^ Centre for Tropical Medicine and Global Health, Nuffield Department of Medicine, University of Oxford, United Kingdom.

^3^ International Centre of Insect Physiology and Ecology, Human Health Theme, Nairobi, Kenya

^4^ College of Veterinary Medicine, Animal Resources and Biosecurity, Makerere University, Kampala, Uganda

^5^ Institute of Microbiology, Infectious Diseases and Immunology, Charité University Medicine, Berlin, Germany

^6^ Institute for Parasitology and Tropical Veterinary Medicine, Freie Universität Berlin, Germany

## Spearman ccorrelation of individual flea counts and tungiasis associated chronic symptoms.

|  |  | Live fleas | Dead fleas | Manipulated lesions | Clusters | Intensity score | Hyper- keratosis | peri-ungual hyperkeratosis | Deformed nails | Lost nails |
| --- | --- | --- | --- | --- | --- | --- | --- | --- | --- | --- |
| Live fleas | rho | 1.0000 |  |  |  |  |  |  |  |  |
| Dead fleas | rho | 0.5268 | 1.0000 |  |  |  |  |  |  |  |
|  | p value | 0.0000 |  |  |  |  |  |  |  |  |
| Manipulated lesions | rho | 0.3261 | 0.3983 | 1.0000 |  |  |  |  |  |  |
|  | p value | 0.0000 | 0.0000 |  |  |  |  |  |  |  |
| Clusters | rho | 0.3928 | 0.3567 | 0.1992 | 1.0000 |  |  |  |  |  |
|  | p value | 0.0000 | 0.0000 | 0.0000 |  |  |  |  |  |  |
| Intensity score | rho | 0.7866 | 0.7996 | 0.5592 | 0.6132 | 1.0000 |  |  |  |  |
|  | p value | 0.0000 | 0.0000 | 0.0000 | 0.0000 |  |  |  |  |  |
| Hyperkeratosis | rho | 0.3816 | 0.5906 | 0.2235 | 0.4225 | 0.5716 | 1.0000 |  |  |  |
|  | p value | 0.0000 | 0.0000 | 0.0000 | 0.0000 | 0.0000 |  |  |  |  |
| peri-ungual hyperkeratosis | rho | 0.4647 | 0.4059 | 0.2679 | 0.425 | 0.5455 | 0.3678 | 1.0000 |  |  |
|  | p value | 0.0000 | 0.0000 | 0.0000 | 0.0000 | 0.0000 | 0.0000 |  |  |  |
| Deformed nails | rho | 0.5224 | 0.6093 | 0.3042 | 0.4661 | 0.6844 | 0.5648 | 0.6261 | 1.0000 |  |
|  | p value | 0.0000 | 0.0000 | 0.0000 | 0.0000 | 0.0000 | 0.0000 | 0.0000 |  |  |
| Lost nails | rho | 0.2893 | 0.2429 | 0.2198 | 0.2023 | 0.3394 | 0.2041 | 0.1938 | 0.1894 | 1.0000 |
|  | p value | 0.0000 | 0.0000 | 0.0000 | 0.0000 | 0.0000 | 0.0000 | 0.0000 | 0.0000 |  |
